# Employment and health after retirement in Japanese men

**DOI:** 10.2471/BLT.18.215764

**Published:** 2018-09-27

**Authors:** Shohei Okamoto, Tomonori Okamura, Kohei Komamura

**Affiliations:** a Graduate School of Economics, Keio University, 2-15-45 Mita, Minato-ku, Tokyo, Japan.; b Department of Preventive Medicine and Public Health, Keio University, Tokyo, Japan.; c Faculty of Economics, Keio University, Tokyo, Japan.

## Abstract

**Objective:**

To estimate the average treatment effect of working past the current retirement age on the health of Japanese men.

**Methods:**

We used publicly available data from the National Survey of Japanese Elderly, extracting a sample of 1288 men who were 60 years or older. Survey respondents were followed-up for at most 15 years for the onset of four health outcomes: death, cognitive decline, stroke and diabetes. By using the propensity score method, we adjusted for the healthy worker effect by incorporating economic, sociodemographic and health data in the form of independent variables. By calculating the differences in times to a health outcome between those in employment and those not employed, we estimated the average treatment effects on health of being in paid work past retirement age.

**Findings:**

Compared with those not employed, those in employment lived 1.91 years longer (95% confidence interval, CI: 0.70 to 3.11), had an additional 2.22 years (95% CI: 0.27 to 4.17) before experiencing cognitive decline, and had a longer period before the onset of diabetes and stroke of 6.05 years (95% CI: 4.44 to 7.65) and 3.35 years (95% CI: 1.42 to 5.28), respectively. We also observed differences between employees and the self-employed: the self-employed had longer life expectancies than employees. In terms of years to onset of diabetes or stroke, however, we only observed significant benefits to health of being an employee but not self-employed.

**Conclusion:**

Our study found that being in employment past the current age of retirement had a positive impact on health.

## Résumé

**Objectif:**

Estimer l'effet moyen du traitement « Travailler après l'âge actuel de la retraite » sur la santé des hommes japonais.

**Méthodes:**

Nous avons utilisé les données publiquement disponibles d'une enquête nationale menée auprès de personnes âgées japonaises, en extrayant un échantillon de 1 288 hommes de 60 ans ou plus. Les personnes ayant participé à cette enquête ont été suivies pendant au maximum 15 ans pour détecter la survenue de quatre événements de santé: décès, déclin cognitif, incident ischémique et diabète. En utilisant la méthode du score de propension, nous avons procédé à un ajustement pour compenser l'effet du travailleur en bonne santé, en incorporant des données économiques, sociodémographiques et des données de santé, sous la forme de variables indépendantes. En calculant les différences constatées -entre les hommes qui travaillaient et ceux qui ne travaillaient pas- dans les délais d'apparition de ces événements de santé, nous avons fait une estimation des effets moyens sur la santé du fait d'avoir un travail rémunéré après l'âge de la retraite.

**Résultats:**

Comparativement aux hommes qui ne travaillaient pas, les hommes qui avaient un travail ont vécu 1,91 année de plus (intervalle de confiance -IC- à 95%: de 0,70 à 3,11), ils ont bénéficié de 2,22 années supplémentaires avant de connaître un déclin cognitif (IC à 95%: de 0,27 à 4,17) et ils ont bénéficié d'une différence de délai encore plus importante avant l'apparition de problèmes de diabète ou d'incidents ischémiques, à savoir 6,05 années (IC à 95%: de 4,44 à 7,65) et 3,35 années (IC à 95%: de 1,42 à 5,28) respectivement. Nous avons également constaté des différences entre les personnes salariées et les travailleurs indépendants, avec une espérance de vie plus élevée chez les travailleurs indépendants. Néanmoins, concernant le délai d'apparition du diabète ou d'incidents ischémiques, nous avons uniquement observé des avantages significatifs pour la santé au fait d’être salarié, mais pas au fait d’être travailleur indépendant.

**Conclusion:**

Notre étude révèle que le fait de travailler après l'âge de la retraite actuellement en vigueur a un impact positif sur la santé.

## Resumen

**Objetivo:**

Estimar el efecto medio del tratamiento de trabajar después de la edad de jubilación actual en la salud de los hombres japoneses.

**Métodos:**

Se utilizaron los datos disponibles públicamente de la Encuesta Nacional a la Tercera edad de Japón, de donde se obtuvo una muestra de 1288 hombres de 60 años o más. A los encuestados se les realizó un seguimiento durante un máximo de 15 años de la aparición de cuatro resultados de salud: muerte, deterioro cognitivo, accidentes cerebrovasculares y diabetes. Mediante el uso del método de puntuación de propensión, se ajustó el efecto del trabajador sano mediante la incorporación de datos económicos, sociodemográficos y de salud en forma de variables independientes. Mediante el cálculo de las diferencias temporales hasta un resultado de salud entre los que trabajan y los que no, se estimaron los efectos del tratamiento promedio sobre la salud de estar en un trabajo remunerado después de la edad de jubilación.

**Resultados:**

En comparación con los que no trabajan, los trabajadores vivían 1,91 años más (95 % intervalo de confianza, IC: 0,70 a 3,11), tenían 2,22 años adicionales (IC del 95 %: 0,27 a 4,17) antes de experimentar un declive cognitivo y un periodo más largo antes de la aparición de diabetes y accidentes cerebrovasculares de 6,05 (IC del 95 %: 4,44 a 7,65) y 3,35 años (IC del 95 %: 1,42 a 5,28), respectivamente. También se observaron diferencias entre los trabajadores por cuenta ajena y por cuenta propia: los últimos tenían una esperanza de vida más larga que los primeros. Sin embargo, en términos de años hasta la aparición de diabetes o accidentes cerebrovasculares, solo se observaron beneficios significativos para la salud en trabajar por cuenta ajena, pero no por cuenta propia.

**Conclusión:**

El estudio concluyó que tener trabajo después de la edad de jubilación actual tuvo un impacto positivo en la salud.

## ملخص

**الغرض:**

تقدير ​​التأثير المتوسط للعلاج بعد سن التقاعد الحالي على صحة الرجال اليابانيين.

**الطريقة:**

اعتمدنا على البيانات المتاحة للعامة من المسح الوطني للمسنين اليابانيين، حيث استخرجنا عينة من 1288 رجلاً تبلغ أعمارهم 60 عامًا أو أكثر. وتمت متابعة المشاركين في المسح لمدة 15 سنة على الأكثر لمتابعة ظهور أربع نتائج صحية: الموت، والتدهور الإدراكي، والسكتة الدماغية، والسكري. وباستخدام طريقة النزعة الطبيعية، قمنا بتعديل تأثير العامل الصحي من خلال دمج البيانات الاقتصادية والاجتماعية والسكانية والصحية في شكل متغيرات مستقلة. وباحتساب الاختلافات في أوقات النتيجة صحية بين أولئك الذين مازالوا يعملون، وأولئك الذين لا يعملون، فقد قمنا بتقدير التأثير المتوسط للعلاج على الصحة في العمل المدفوع الأجر بعد سن التقاعد.

**النتائج:**

مقارنة مع أولئك الذين لا يعملون، عاش الذين يعملون فترة أطول بـ 1.91 سنة (فاصل الثقة 95%: 0.70 إلى 3.11)، ومر عليهم 2.22 سنة إضافية (فاصل الثقة 95%: 0.27 إلى 4.17) قبل أن يتعرضوا للتدهور الإدراكي، كما كان لديهم فترة أطول قبل ظهور مرض السكري والسكتة الدماغية وهي 6.05 سنوات (فاصل الثقة 95%: 4.44 إلى 7.65)، و3.35 سنوات (فاصل الثقة 95%: 1.42 إلى 5.28) على الترتيب. كما لاحظنا أيضا الاختلافات بين الموظفين وأصحاب المهن الحرة: حيث كان لدى أصحاب المهن الحرة متوسط ​​حياة أطول من الموظفين. إلا أنه فيما يتعلق بسنوات الإصابة بمرض السكري أو السكتة الدماغية، لاحظنا فقط مزايا ملموسة لدى الموظفين وليس لدى أصحاب المهن الحرة.

**الاستنتاج:**

وجدت دراستنا أن التواجد في العمل بعد سن التقاعد الحالي كان له تأثير إيجابي على الصحة.

## 摘要

**目的:**

旨在评估日本男性在当前退休年龄后参与就业对健康的平均疗效。

**方法:**

我们采用日本老年人全国性调查 (National Survey of the Japanese Elderly) 的公开数据，提取了由 1288 名 60 岁或以上男性组成的样本。对参与调查的受访者的四种健康结果，即死亡、认知能力下降、中风和糖尿病，进行了至多 15 年的随访。我们使用倾向性评分法，以独立变量的形式纳入经济、社会人口统计学和健康数据，对健康工人效应进行调整。通过计算就业者与未就业者健康结果之间的时间差异，我们估算了当前退休年龄后参与有偿工作对健康的平均疗效。

**结果:**

与未就业者相比，就业者的寿命延长了 1.91 年（95％ 置信区间，CI：0.70 至 3.11)，认知能力下降推迟了 2.22 年（95% 置信区间，CI：0.27 至 4.17)，糖尿病和中风推迟的时间更久，分别推迟了 6.05 年（95% 置信区间，CI：4.44 至 7.65）和 3.35 年（95% 置信区间，CI：1.42 至 5.28）。我们还观察到雇员与自雇者之间的差异：自雇者的预期寿命比雇员更长。然而，我们仅发现雇员较自雇者在糖尿病或中风发病年限上有显著优势。

**结论:**

研究发现，在当前退休年龄后参与就业对健康产生了积极影响。

## Резюме

**Цель:**

Оценить полезное влияние трудовой занятости после достижения текущего пенсионного возраста на здоровье японских мужчин.

**Методы:**

Использовались общедоступные данные Национального исследования пожилых японцев, в ходе которого была получена выборка из 1288 мужчин в возрасте 60 лет и старше. Участники исследования наблюдались на протяжении максимум 15 лет до наступления четырех исходов в отношении здоровья: смерти, снижения когнитивных способностей, инсульта и диабета. Используя метод оценки предрасположенности, исследователи скорректировали производительность здорового работника с учетом экономических, социально-демографических и медицинских показателей в виде независимых переменных. Путем вычисления разницы во времени до наступления исходов в отношении здоровья между работающими и неработающими пенсионерами авторы оценили полезное влияние трудовой занятости на здоровье после достижения пенсионного возраста.

**Результаты:**

В сравнении с неработающими пенсионерами продолжительность жизни работающих пенсионеров составила на 1,91 года больше (95%-й ДИ: 0,70–3,11), начало снижения когнитивных способностей наступало на 2,22 года позже (95%-й ДИ: 0,27–4,17), у них был более длительный период до развития диабета и инсульта — 6,05 года (95%-й ДИ: 4,44–7,65) и 3,35 года (95%-й ДИ: 1,42–5,28) соответственно. Исследователи также наблюдали различия между работающими по найму и самостоятельно занятыми пенсионерами: у самостоятельно занятых была большая продолжительность жизни, чем у работающих по найму. Тем не менее пенсионеры, работающие по найму, продемонстрировали лучшие результаты в отношении количества лет до развития диабета или инсульта по сравнению с самостоятельно занятыми пенсионерами.

**Вывод:**

Исследование показало, что сохранение трудовой занятости после достижения текущего пенсионного возраста оказывает положительное влияние на здоровье.

## Introduction

In response to an ageing population and the associated financial challenges, many countries have introduced extensions to working life; however, research on the resultant effect on health is currently insufficient. In Japan, the age of income-related pension eligibility is gradually being increased from 60 years in 2013 by 1 year, every 3 years, to reach 65 years in 2030 for both men and women.^1^ Policies which set minimum mandatory retirement age (usually 60 years, but can vary from one company to another) have recently been updated, and workers are now free to continue in paid employment past this age if they desire.^2^

Although several studies have already investigated the association between health and retirement,^3^^–^^11^ they failed to identify causal relationships. As the age of retirement and condition of health can be interdependent, health selection exists;^12^^,^^13^ causal inference approaches are therefore required to measure the impact of working past current retirement age on health. A limited number of empirical studies have been conducted to determine causal impacts, but results are inconsistent. Some studies report negative effects on self-rated health, cognitive functioning and depressive syndrome because of retirement;^14^^–^^17^ however, other studies have shown that retirement can lead to improvements in health as indicated by the numbers diagnosed with chronic conditions, activity limitations and self-rated health.^18^^,^^19^ Only a few studies have accounted for the healthy worker effect when assessing the health consequences of working past retirement age. These studies did not observe any health benefits.^17^^,^^20^

We contribute to this area of study in two ways. We first expand the health outcomes examined compared with those in previous publications,^3^^–^^6^^,^^9^^,^^17^^–^^19^^,^^21^ which have generally only considered self-rated physical or mental health, activity limitations and cognitive decline, to consider the three additional health outcomes of death, stroke and diabetes. Analyses of stroke and diabetes are particularly beneficial in investigating the rising costs of health and long-term care in the Japanese population.^22^ Second, we quantify the effect of working past retirement age in terms of delay in years to the onset of these health outcomes. We believe this approach provides greater insight into healthy life expectancy compared with studies that investigate the likelihood of certain diseases by estimating hazard or odds ratios.

## Methods

### Data

We retrieved data from the National Survey of the Japanese Elderly,^23^ a six-wave longitudinal prospective survey of a sample of Japanese men and women aged 60 years or older. The survey began with a population of 2200 in 1987, and was subsequently supplemented in later waves in 1990, 1993, 1996, 1999 and 2002 (Table 1). The sample was extracted from the Basic Resident Registration System, administered by the Japanese government, by stratified two-stage random sampling. Municipalities were first selected based on regions and population size, and then survey participants were randomly selected in a manner consistent with the age distribution of Japan; face-to-face interviews were performed at each wave. We have focused our analysis on men only, since labour force participation rates were lower for women (50.1% in 1998 versus 77.3% for men).^24^ Response rates at each wave are listed in Table 1.

**Table 1:** National Survey of the Japanese Elderly response rates of men and women 60 years or older, 1987–2002

Wave (year)	Continued^a^ or additional	*n*	No. of participants providing valid responses (%)
1 (1987)	–	3288	2200 (66.9)
2 (1990)	Continued	2200	1671 (76.0)
	Additional	580	366 (63.1)
3 (1993)	Continued	2441	1864 (76.4)
4 (1996)	Continued	2226	1549 (69.6)
	Additional	1210	898 (74.2)
5 (1999)	Continued	2969	2077 (70.0)
	Additional	2000	1405 (70.3)
6 (2002)	Continued	4336	2823 (65.1)

^a^ Number of responses in continued waves takes account of the deaths of some respondents between waves.

### Health outcomes

We used publicly available data, in which each survey participant was followed-up for the four health outcomes at each wave for at most 15 years. Information regarding the death of a participant, including date, was obtained from the Basic Resident Registration System. Assessment of the three other outcomes (cognitive function, stroke and diabetes) was made at each wave.

Cognitive functioning was assessed based on the short portable mental status questionnaire.^25^ This memory test comprises nine questions: the respondent’s address; date of interview; day of interview; mother’s maiden name; name of the current prime minister; name of the previous prime minister; a simple calculation; the subject’s birthday; and the subject’s age. This is a similar approach to standard memory tests used in the field of cognitive science.^26^^,^^27^ If the respondent made more than five mistakes, their cognitive functioning was recorded as moderately declined.^25^ If a participant was not actually able to complete an interview due to cognitive decline, they were assessed as having a cognitive impairment.

The onset of stroke or diabetes was determined according to self-reported symptoms in the survey questionnaire. The questionnaire also recorded information on whether disease onset was based on a diagnosis by a medical professional or by the participant.

### Independent variables

We based the values of the independent variables used to adjust for confounders on the baseline (i.e. first) survey responses for each participant. The most important reason for this was that some participants had undergone a decline in cognitive functioning, reducing the reliability of their answers. Individuals older than 75 years at baseline were excluded from the analysis, since only 48 men were in employment.

We defined being in employment as a binary variable of value 1 if individuals were receiving a wage from their job at baseline, including all contract types (i.e. as a full- or part-time employee or being self-employed), and 0 if not employed.^20^ We included demographic information in the form of age and marital status, defining marital status as a binary variable of value 1 if married and 0 if not married (including those bereaved or divorced). We considered socioeconomic data in terms of home ownership (1 if people owned their own homes, 0 otherwise), equalized household income (the household income divided by the square root of the number of household members), educational attainment (proportion within one of four categories of years in education)^28^ and proportion within one of five longest-held employment types. Following Kan,^23^ a respondent’s longest-held occupation type was classified as either: professional or administrative; service or clerical; agriculture, forestry or fisheries; manual labour; or self-employed. We included condition of health in the model in terms of whether a smoker (1 if yes, 0 if not), self-rated health (1 for poor health, 0 otherwise) and body mass index (kg/m). To attempt to counteract the healthy worker effect, individuals of abnormal weight (i.e. a mean body mass index ± 3 standard deviations from the mean) were excluded from the analysis.

### Propensity score method

We adopted the propensity score method to estimate the effects on health of being in employment when older than 60 years. The propensity score method was originally developed to investigate the presence of a causal inference in non-experimental (i.e. observational and therefore non-randomized) surveys, while attempting to reduce the bias due to confounding variables;^29^ previous investigations have demonstrated its use in dealing with potential observed endogeneity (i.e. outcomes which are not independent of baseline characteristics).^30^ We have applied the propensity score method here to account for the fact that individuals in poorer health may have retired earlier.^12^^,^^13^

We calculated the propensity score using Stata software, version 14.2 (StataCorp LLC, College Station, United States of America), as the conditional probability of being in employment or not at baseline. This conditional probability was obtained by logistic regression using the covariates describing the economic, sociodemographic and health characteristics of survey respondents. We then used the inverse probability of being in paid work (i.e. propensity score) to balance the covariates between those in employment and those not employed.

Incorporating the propensity score, we used survival analysis to estimate time to a particular health outcome from the first survey while assuming a Weibull distribution. By calculating the differences in times to an outcome for both those in employment and those not employed, we estimated the weighted average treatment effects on health of being in paid work past retirement age.

## Results

We retrieved data for 1288 men that were eligible for analysis on an available-case basis. The health outcome data for the participating men are provided in Table 2. Men not employed had a shorter time to death than men with employment (7.66 years versus 9.31 years; *P* < 0.001). We observed similar outcome for cognitive decline: men not employed had 7.58 years to onset, while men with employment had 11.20 years (*P* = 0.003). Men not employed had 5.84 years to the onset of stroke, which was less than the 8.03 years for men with employment (*P* = 0.001). Time to onset for diabetes was not significant between the groups (4.06 years for men not employed versus 3.96 years for men with employment; *P* = 0.319; Table 3).

**Table 2:** Observed health outcomes of male participants of the National Survey of the Japanese Elderly after a 15-year follow-up, 1987–2002

Health outcome	Men not employed				Men in employment			*P*^c^ for difference in % affected	*P*^d^ for difference in onset time
	*n*^a^	No. affected (%)	Average time to onset, years^b^		*n*^a^	No. affected (%)	Average time to onset, years		
Death	640	217 (34)	7.66		644	126 (20)	9.31	< 0.001	< 0.001
Cognitive decline	640	36 (6)	7.58		645	18 (3)	11.20	< 0.001	0.003
Stroke	494	97 (20)	5.84		499	68 (14)	8.03	0.007	0.001
Diabetes	454	105 (23)	4.06		462	106 (23)	3.96	0.878	0.319

^a^ Sample size for available-case analysis.

^b^ Average time is between the first survey and onset.

^c^ *χ^2^* test.

^d^ Welch *t*-test.

Note: We used a sample of 1288 men who were 60 years or older.

**Table 3:** Independent variables used to estimate average treatment effect of working past retirement age for Japanese men, 1987–2002

Independent variable	Men not employed *n* = 643	Men in employment *n* = 645	*P*^a,b^
**Mean equalized household income, × 10 000 yen (SD)**	199 (133)	323 (250)	< 0.001
**Owning own home, mean % (SD)**	82 (38)	85 (36)	0.162
**Educational attainment category in years, mean % (SD)**			
0–7	12 (32)	7 (25)	0.003
8–9	41 (49)	43 (50)	
10–11	21 (40)	18 (39)	
> 12	27 (44)	32 (47)	
**Married, mean % (SD)**	90 (30)	94 (23)	0.003
**Smokers, mean % (SD)**	47 (50)	46 (50)	0.508
**Self-reporting poor health, mean % (SD)**	17 (38)	07 (26)	< 0.001
**Mean body mass index, kg/m (SD)**	22 (3)	22 (3)	0.098
**Mean age, years (SD)**	68 (5)	65 (5)	< 0.001
**Longest-held employment type, mean % (SD)**			
Professional or administrative	26 (44)	11 (31)	< 0.001
Service or clerical	17 (38)	6 (23)	
Agriculture, forestry or fisheries	6 (23)	3 (17)	
Manual labour	45 (50)	13 (34)	
Self-employment	7 (25)	67 (47)	

SD: standard deviation.

^a^ *P*-values of differences in variables between those not employed and those in employment. There is only one *P-*value for each of the combined education and employment categories because we only investigated whether a proportion of some category was different between the two groups.

^b^ Welch *t* test for continuous variables and χ^2^ test for categorical variables.

Note: Inconsistencies arise in some values due to rounding.

The differences in values of independent variables between those in employment and those not employed are shown in Table 3. Participants in employment had higher incomes than those who were not employed (whose income was mostly in the form of pension benefits). Subjective feelings of being in poor health were significantly higher for individuals not in employment (average: 17%) compared with those who were employed (average: 7%). This example highlights how individuals not in employment may have been in that position due to poor health, which would reinforce the healthy worker effect unless adjusted for. We observed another major difference in the longest-held occupation type; the proportion of self-employed among individuals still working was much higher than those no longer in employment (averages of 68% versus 7%).

Table 4 provides the estimated average treatment effects of being in employment past retirement age compared with not being employed on the four different health outcomes. We cannot reject the null hypotheses that the covariates were balanced between the employed and not employed groups; this implies that confounders have been adequately adjusted for.

**Table 4:** Estimated average treatment effects of being in employment past retirement age on health outcomes for Japanese men, 1987–2002

Health outcome^a^	Men not employed,^b^ *n*	Men in employment,^b^ *n*	Time to onset for those not employed, years^c^	Average treatment effect of being in employment,^d^ years (95% CI)	Balancing^e^ *P*
Death	640	644	9.40	1.91 (0.70 to 3.11)	0.245
Cognitive decline	640	645	10.58	2.22 (0.27 to 4.17)	0.284
Stroke	494	499	11.08	3.35 (1.42 to 5.28)	0.412
Diabetes	454	462	8.52	6.05 (4.44 to 7.65)	0.518

CI: confidence interval.

^a^ The maximum years of follow-up was 15 years after the first survey.

^b^ Sample size for available-case analysis.

^c^ Modelled by propensity score method.

^d^ Average treatment effect estimated via survival analysis with inverse probability weighting.

^e^ Null hypothesis is that covariates (economic, sociodemographic and health characteristics as described in Table 3) are balanced.

Note: We used men not employed as control group.

There were significant differences in all four outcomes between the two groups: participants in employment were likely to experience longer periods of good health regarding all four outcomes compared with those not employed. On average, individuals not employed died 9.40 years after their baseline surveys; those in employment died 1.91 years later (95% confidence interval, CI: 0.70 to 3.11). Participants not employed underwent cognitive decline 10.58 years after their baseline; those in paid work did not experience cognitive decline for another 2.22 years (95% CI: 0.27 to 4.17). Being in employment prolonged the disease-free period for stroke by 3.35 years (95% CI: 1.42 to 5.28) beyond the 11.08 years of onset among participants not employed. The onset of diabetes among individuals not employed began 8.52 years after baseline; for those in employment, it occurred 6.05 years later (95% CI: 4.44 to 7.65).

A substantial proportion of participants were self-employed. We therefore performed another analysis, separating those in employment into employees and the self-employed, and compared with those not employed (Table 5). Life expectancies were significantly longer among both employees (average treatment effect: 1.88 years; 95% CI: 0.35 to 3.73) and the self-employed (average treatment effect: 2.74 years; 95% CI: 1.30 to 4.17) compared with those not employed (onset of death at 9.17 years). Significant improvements in morbidity in terms of diabetes and stroke were only observed among employees, however, and not the self-employed. In comparisons with those not employed, extensions to healthy life were observed for employees for diabetes (average treatment effect: 6.30 years; 95% CI: 5.00 to 7.61) and stroke (average treatment effect: 3.90 years; 95% CI: 1.67 to 6.13). Health benefits for the self-employed for diabetes (average treatment effect: 0.19; 95% CI: –1.72 to 2.09) and stroke (average treatment effect: 1.64; 95% CI: –0.87 to 4.15) were not observed, however. We did not observe any significant difference between employees and the self-employed in terms of years to onset of cognitive decline.

**Table 5:** Estimated average treatment effects of being an employee or being self-employed past retirement age on health outcomes for Japanese men, 1987–2002

Health outcome	Men not employed,^a^ *n*	Employees,^a^ *n*	Self-employed men,^a^ *n*	Time to onset for those not employed, years^b^	Average treatment effect of being an employee,^c^ years (95% CI)	Average treatment effect of being self-employed,^c^ years (95% CI)
Death	640	83	561	9.17	1.88 (0.35 to 3.73)	2.74 (1.30 to 4.17)
Cognitive decline	640	84	561	10.57	1.16 (–1.91 to 4.23)	1.51 (–0.18 to 3.19)
Stroke	494	59	440	10.74	3.90 (1.67 to –6.13)	1.64 (–0.87 to 4.15)
Diabetes	454	58	404	8.56	6.30 (5.00 to 7.61)	0.19 (–1.72 to 2.09)

CI: confidence interval.

^a^ Sample size for available-case analysis.

^b^ Modelled by propensity score method.

^c^ Average treatment effect estimated via survival analysis with inverse probability weighting.

Note: As a control group, we used men not employed.

## Discussion

In line with studies which evaluated the health impacts of retirement in Japan and the United States,^14^^–^^17^ our estimates of average treatment effects imply that being in paid work past the current age of retirement has positive effects on health. 

There are three main interpretations of the results. First, according to the human capital model,^31^ people may be incentivized to invest in health for the sake of increasing or maintaining their productivity and wages. If individuals have a problem with their health, they may experience presenteeism or absenteeism; they may even have to retire earlier than planned as a result of poor health. Second, studies have reported^32^^–^^34^ that the social participation and networks that accompany most jobs can enhance health. Having increased social relationships is directly linked to improved physical and mental health outcomes, for example avoiding stress and depression.^35^^,^^36^ Third, the latent functions of employment, such as social status and self-respect, also contribute positively to health outcomes.^37^^–^^39^ We did not investigate which of these three hypotheses has the greatest effect; these hypotheses need to be examined in detail in further studies before they can be usefully incorporated by policy-makers.

In an additional analysis, we examined the difference between effects on health for employees and the self-employed. Employees had better health than the self-employed regarding estimated onset to stroke and diabetes, whereas the estimated life expectancy of the self-employed was longer. One of the most significant explanations is that, in accordance with industrial safety and health law, employers in Japan are required to provide annual health check-ups; these are mainly focused on cardiovascular risk factors (e.g. hypertension and diabetes). Health check-up attendance among the self-employed is very low compared with employees, as these must be organized by the individuals.^40^ In addition, since the wages of employees can be determined by their employers according to their performance and human capital,^31^ which are in turn affected by their health, employees may be more motivated to manage their health than the self-employed. Policies which encourage the self-employed to seek and maintain better health are therefore necessary, such as financial incentives.^41^

Our study has some limitations. First, the case ascertainment of diabetes and stroke was based on self-reported symptoms, whereas death and cognitive decline were measured from demographic statistics and an established test. The questionnaire did record whether participants had been diagnosed with diabetes or as having had a stroke by a medical professional, but the source of diagnosis was not considered in our analysis. Caution should therefore be exercised when interpreting the results regarding these two health outcomes.

Second, our sample set comprised respondents of the National Survey of the Japanese Elderly, all of whom had survived to age 60 years or older, perhaps contributing to the healthy worker effect. We attempted to overcome this problem by using the propensity score method with survival analysis; by incorporating differences in health, measured from variables including body mass index and self-rated health, we have considered the consequences of behavioural health. In addition, the survival analysis method is focused on the onset of new health outcomes; those who were already unhealthy, that is, with a history of any of the health outcomes observed here, were excluded from the analysis.

Third, we did not consider changes in employment status. In an extreme case, a respondent could have changed their status from employed to not employed immediately after the baseline survey. There may also have been changes to health related to the total time in employment during the follow-up period. Further, beyond distinguishing between being employed or self-employed, we did not consider the frequency of work, job security or availability of any fringe benefits; such factors can also have an effect on health,^42^ and future investigations should consider these.

In conclusion, this empirical study implies that being in paid work in later life is beneficial for both mortality and some outcomes of morbidity, prolonging healthy periods. Although more research is needed to investigate the effects of transition of employment status and other precise health outcomes, our estimates of average treatment effects indicate that extending working lives has benefits to health. Policies designed for longer working lives should incorporate appropriate management of lifelong physical and mental health practices and conditions.

## References

[1] The Pension Group of the Fourth Social Security Council. [About the pension age.] Tokyo: Ministry of Health, Labour and Welfare; 2011. [Japanese]. Available from: https://www.mhlw.go.jp/stf/shingi/2r9852000001r5uy.html [cited 2018 Sep 17].

[2] General survey on working conditions Tokyo: Ministry of Health, Labour and Welfare; 2016. [Japanese]. Available from: https://www.mhlw.go.jp/toukei/itiran/roudou/jikan/syurou/17/ [cited 2018 Sep 17].

[3] Moon JR, Glymour MM, Subramanian SV, Avendaño M, Kawachi I. Transition to retirement and risk of cardiovascular disease: prospective analysis of the US health and retirement study. Soc Sci Med. 2012 8;75(3):526–30. 10.1016/j.socscimed.2012.04.004 22607954 PMC3367095

[4] Westerlund H, Vahtera J, Ferrie JE, Singh-Manoux A, Pentti J, Melchior M, et al. Effect of retirement on major chronic conditions and fatigue: French GAZEL occupational cohort study. BMJ. 2010 11 23;341(1):6149. 10.1136/bmj.c6149 21098617 PMC2990862

[5] Westerlund H, Kivimäki M, Singh-Manoux A, Melchior M, Ferrie JE, Pentti J, et al. Self-rated health before and after retirement in France (GAZEL): a cohort study. Lancet. 2009 12 5;374(9705):1889–96. 10.1016/S0140-6736(09)61570-1 19897238

[6] Jokela M, Ferrie JE, Gimeno D, Chandola T, Shipley MJ, Head J, et al. From midlife to early old age: health trajectories associated with retirement. Epidemiology. 2010 5;21(3):284–90. 10.1097/EDE.0b013e3181d61f53 20220519 PMC3204317

[7] van Solinge H. Health change in retirement: a longitudinal study among older workers in the Netherlands. Res Aging. 2007;29(3):225–56. 10.1177/0164027506298223

[8] Butterworth P, Gill SC, Rodgers B, Anstey KJ, Villamil E, Melzer D. Retirement and mental health: analysis of the Australian national survey of mental health and well-being. Soc Sci Med. 2006 3;62(5):1179–91. 10.1016/j.socscimed.2005.07.013 16171915

[9] Mein G, Martikainen P, Hemingway H, Stansfeld S, Marmot M. Is retirement good or bad for mental and physical health functioning? Whitehall II longitudinal study of civil servants. J Epidemiol Community Health. 2003 1;57(1):46–9. 10.1136/jech.57.1.46 12490648 PMC1732267

[10] Fisher GG, Chaffee DS, Sonnega A. Retirement timing: a review and recommendations for future research. Work Aging Retire. 2016;2(2):230–61. 10.1093/workar/waw001

[11] Smeaton D, McKay S. Working after state pension age: quantitative analysis. London: Department for Work and Pensions; 2003.

[12] McGarry K. Health and retirement: do changes in health affect retirement expectations? J Hum Resour. 2004;XXXIX(3):624–48. 10.3368/jhr.XXXIX.3.624

[13] Hagan R, Jones AM, Rice N. Health shocks and the hazard rate of early retirement in the ECHP. Schweiz Z Volkswirtsch Stat. 2008;144(3):323–35.

[14] Kajitani S. Working in old age and health outcomes in Japan. Japan World Econ. 2011;23(3):153–62. 10.1016/j.japwor.2011.06.001

[15] Kajitani S, Sakata KEI, McKenzie C. Occupation, retirement and cognitive functioning. Ageing Soc. 2017;37(8):1568–96. 10.1017/S0144686X16000465

[16] Bonsang E, Adam S, Perelman S. Does retirement affect cognitive functioning? J Health Econ. 2012 5;31(3):490–501. 10.1016/j.jhealeco.2012.03.005 22538324

[17] Calvo E, Sarkisian N, Tamborini CR. Causal effects of retirement timing on subjective physical and emotional health. J Gerontol B Psychol Sci Soc Sci. 2013 1;68(1):73–84. 10.1093/geronb/gbs097 23149431

[18] Hessel P. Does retirement (really) lead to worse health among European men and women across all educational levels? Soc Sci Med. 2016 2;151:19–26. 10.1016/j.socscimed.2015.12.018 26773290

[19] Coe NB, Zamarro G. Retirement effects on health in Europe. J Health Econ. 2011 1;30(1):77–86. 10.1016/j.jhealeco.2010.11.002 21183235 PMC3972912

[20] Di Gessa G, Corna LM, Platts LG, Worts D, McDonough P, Sacker A, et al. Is being in paid work beyond state pension age beneficial for health? Evidence from England using a life-course approach. J Epidemiol Community Health. 2017 5;71(5):431–8. 10.1136/jech-2016-208086 27940656 PMC5484027

[21] Hyde M, Ferrie J, Higgs P, Mein G, Nazroo J. The effects of pre-retirement factors and retirement route on circumstances in retirement: findings from the Whitehall II study. Ageing Soc. 2004;24(02):279–96. 10.1017/S0144686X03001624

[22] Patient survey 2014. Disease and injury. Tokyo: Ministry of Health, Labour and Welfare; 2014. [Japanese]. Available from: https://www.mhlw.go.jp/toukei/saikin/hw/kanja/14/index.html [cited 2018 Sep 17].

[23] Kan M. Econometric analysis of disparities in health among Japanese elderly: with an emphasis on the effects of the Elderly Health Care System. Japanese J Health Econ Policy. 2009;20(2):85–108. [Japanese]

[24] The present status of gender equality and measures. Tokyo: Gender Equality Cabinet Bureau Office; 1998. Available from: http://www.gender.go.jp/english_contents/about_danjo/whitepaper/plan2000/1999/p1c201.html [cited 2018 Sep 17].

[25] Pfeiffer E. A short portable mental status questionnaire for the assessment of organic brain deficit in elderly patients. J Am Geriatr Soc. 1975 10;23(10):433–41. 10.1111/j.1532-5415.1975.tb00927.x 1159263

[26] Folstein MF, Folstein SE, McHugh PR. “Mini-mental state”: A practical method for grading the cognitive state of patients for the clinician. J Psychiatr Res. 1975 11;12(3):189–98. 10.1016/0022-3956(75)90026-6 1202204

[27] Teng EL, Hasegawa K, Homma A, Imai Y, Larson E, Graves A, et al. The Cognitive Abilities Screening Instrument (CASI): a practical test for cross-cultural epidemiological studies of dementia. Int Psychogeriatr. 1994 Spring;6(1):45–58, discussion 62. 10.1017/S1041610294001602 8054493

[28] [The history of school education system in Japan.] Tokyo: National Institute for Educational Policy Research; 2012. [Japanese]. Available from: https://www.nier.go.jp/04_kenkyu_annai/pdf/kenkyu_01.pdf [cited 2018 Sep 17].

[29] Rosenbaum PR, Rubin DB. The central role of the propensity score in observational studies for causal effects. Biometrika. 1983;70(1):41–55. 10.1093/biomet/70.1.41

[30] Giménez-Nadal JI, Molina JA. Commuting time and household responsibilities evidence using propensity score matching. J Reg Sci. 2016;56(2):332–59. 10.1111/jors.12243

[31] Grossman M. On the concept of health capital and the demand for health. J Polit Econ. 1972;80(2):223–55. 10.1086/259880

[32] Ichida Y, Hirai H, Kondo K, Kawachi I, Takeda T, Endo H. Does social participation improve self-rated health in the older population? A quasi-experimental intervention study. Soc Sci Med. 2013 10;94:83–90. 10.1016/j.socscimed.2013.05.006 23931949

[33] Holt-Lunstad J, Smith TB, Layton JB. Social relationships and mortality risk: a meta-analytic review. PLoS Med. 2010 7 27;7(7):e1000316. 10.1371/journal.pmed.1000316 20668659 PMC2910600

[34] House JS, Landis KR, Umberson D. Social relationships and health. Science. 1988 7 29;241(4865):540–5. 10.1126/science.3399889 3399889

[35] Cohen S. Social relationships and health. Am Psychol. 2004 11;59(8):676–84. 10.1037/0003-066X.59.8.676 15554821

[36] Uchino BN. Social support and health: a review of physiological processes potentially underlying links to disease outcomes. J Behav Med. 2006 8;29(4):377–87. 10.1007/s10865-006-9056-5 16758315

[37] Jahoda M. Employment and unemployment: a social-psychological analysis. Cambridge: Cambridge University Press; 1982.

[38] Bartley M. Unemployment and ill health: understanding the relationship. J Epidemiol Community Health. 1994 8;48(4):333–7. 10.1136/jech.48.4.333 7964329 PMC1059979

[39] Warr PB. Work, unemployment, and mental health. Oxford: Clarendon Press; 1987.

[40] Okamura T, Sugiyama D, Tanaka T, Dohi S. Worksite wellness for the primary and secondary prevention of cardiovascular disease in Japan: the current delivery system and future directions. Prog Cardiovasc Dis. 2014 Mar-Apr;56(5):515–21. 10.1016/j.pcad.2013.09.011 24607016

[41] Okamoto S, Komamura K, Tanabe K, Yokoyama N, Tsukao A, Chijiki S, et al. Who opts out of a project for health promotion with incentives? Empirical research on the effect of rewards to motivate persistence. Nihon Koshu Eisei Zasshi. 2017;64(8):412–21. 28966338 10.11236/jph.64.8_412

[42] Dooley D, Prause JA. The social costs of underemployment: inadequate employment as disguised unemployment. Cambridge: Cambridge University Press; 2004.

